# Excessive fruit consumption during the second trimester is associated with increased likelihood of gestational diabetes mellitus: a prospective study

**DOI:** 10.1038/srep43620

**Published:** 2017-03-08

**Authors:** Wu-Qing Huang, Ying Lu, Ming Xu, Jing Huang, Yi-Xiang Su, Cai-Xia Zhang

**Affiliations:** 1 Department of Medical Statistics and Epidemiology, School of Public Health, Sun Yat-sen University, Guangzhou 510080, China; 2 Guangzhou Center of Disease Control and Prevention, Guangzhou 510440, China; 3 Department of Nutrition, School of Public Health, Sun Yat-sen University, Guangzhou 510080, China

## Abstract

This study aimed to investigate the association between fruit consumption during the second trimester and the occurrence of gestational diabetes mellitus (GDM). A prospective study with 772 female participants was conducted in China from April 2013 to August 2014. Dietary intake was assessed in face-to-face and telephone interviews using a 3-day food record. GDM was ascertained using a standard 75 g 2 hour oral glucose tolerance test. Multivariable logistic regression was used to estimate odds ratios (ORs) and 95% confidence intervals (CIs) after adjustment for various confounders. Of the 772 participants, 169 were diagnosed with GDM during the period under study. Greater total fruit consumption during the second trimester was associated with a higher likelihood of GDM (highest vs. lowest quartile: adjusted OR4.82, 95% CI 2.38 to 9.76). Fruits with a moderate or high glycaemic index (GI) were positively associated with the occurrence of GDM. Fruit subgroups were also categorised by polyphenol content, and tropical-fruit and citrus-fruit consumption was found to be positively related to the occurrence of GDM. These findings suggest that the excessive consumption of fruit, especially fruit with moderate or high GI values, tropical-fruit and citrus-fruit, increases the likelihood of GDM.

The recorded prevalence of gestational diabetes mellitus (GDM) in China has increased sharply, from about 5% to more than 16%, since the implementation of a new method of diagnosing GDM in December 2011^1^,^2^. GDM is associated with an increased risk of adverse pregnancy and perinatal outcomes and long-term adverse health consequences for both mother and child^3^. Therefore, it is urgently necessary to identify risk factors for GDM. Dietary factors are amongst the most important modifiable factors. With the improvement of living standards, fruit consumption in China has drastically increased, to the extent that an appreciable proportion of pregnant women in China today consume fruit to excess^4^. Fruit is abundant in fibre, antioxidants and phytochemicals, which have beneficial health effects^5^,^6^. However, some kinds of fruit also contain high levels of sugar (e.g., fructose), the excessive intake of which is likely to be harmful to human health^7^,^8^. Epidemiological studies have generated mixed results regarding the relationship between fruit consumption and type 2 diabetes (T2D) risk^9^,^10^,^11^,^12^. Although the Nurses’ Health Study (NHS) II investigated the association between pre-pregnancy habitual fruit consumption and GDM risk, the specific effects of fruit consumption during pregnancy have not yet been examined^13^. One study investigated the association between dietary habits and GDM risk among Cantonese women in China. The results revealed a tendency for excessive fruit consumption by Cantonese women during pregnancy and a positive association between the consumption of fruit with a high glycaemic index (GI) and GDM risk^4^.

In addition, the GI and polyphenol content, which have been suggested to be related to blood-glucose metabolism, differ substantially between types of fruit^14^,^15^. One study indicated that fruits with a moderate GI played a protective role in T2D^16^. Meanwhile, interest in polyphenols has increased notably over the past decade due to the discovery of their antioxidant effects and their role in the prevention of several chronic diseases, including diabetes^15^. One study revealed that fruit subgroups categorised according to polyphenol content had different effects on the risk of mortality from cardiovascular disease^17^, which led us to explore the relative association with GDM risk of fruit subgroups divided by polyphenol content.

Dietary habits during pregnancy have been shown to differ dramatically from normal dietary habits for most women. In addition, whilst most women experience morning sickness during the first trimester of pregnancy, the second trimester is characterised by relatively constant dietary habits, which are more representative of diet across the whole gestation period. Therefore, it is appropriate to evaluate the association between fruit consumption during the second trimester and the occurrence of GDM.

This study targeted women in the second trimester of pregnancy (13 to 27 gestational weeks). The aim of the study was to evaluate the influence on GDM of overall fruit consumption and the consumption of fruit subgroups categorised according to GI and polyphenol content.

## Results

### Comparison of baseline characteristics between final cohort and participants lost to follow-up

During the follow-up period (from April 2013 to August 2014), data provided by 772 eligible participants were included in the analysis. As shown in Table 1, the final cohort reported a mean age of 26.01 years. One hundred and sixty-nine (21.9%) of the participating pregnant women received a diagnosis of GDM. The baseline characteristics of the final cohort and the population lost to follow-up were largely similar. However, the pregnant women lost to follow-up had a slightly lower socio-occupational status and education level than those subject to analysis.

### Baseline characteristics of study population

When allocated to quartiles by fruit consumption, the participants who reported higher overall fruit consumption were older (*P* = 0.03) and had a higher intake of energy, carbohydrate and protein (*P* < 0.001). No significant differences were seen in occupation, income level, exercise, smoking habits, alcohol use, family history of diabetes, pre-pregnancy body mass index (BMI) and gestation weight gain between the quartiles (Table 2).

### Excessive total fruit consumption during the second trimester increased the occurrence of GDM

As shown in Table 3, the median amount of fresh fruit consumed by the subjects during the second trimester of pregnancy was 349 g/d. An increase in total fruit consumption during the second trimester was associated with an elevated likelihood of GDM (highest vs. lowest quartile: crude OR, 3.20; 95% CI, 1.83 to 5.60). After adjustment for age, education, occupation, income level, pre-pregnancy BMI, gestational weight gain, family history of diabetes, smoking status and alcohol use in Model 1, a significantly higher likelihoodof GDM was still observed in the third and fourth quartiles for total fruit consumption (OR 2.81; 95% CI 1.47 to 5.36; OR 3.47; 95% CI 1.78 to 6.36, respectively). After adjustment for potential confounding factors in Model 1 plus the consumption of grain, vegetables, meat and fish, the ORs for the lowest to the highest quartiles of fruit consumption were 1.00 (reference), 1.08 (95% CI 0.50 to 2.34), 3.03 (95% CI 1.54 to 5.94) and 4.82 (95% CI 2.38 to 9.76), respectively. When analyses were carried out using continuous variable, a significant positive association was also observed (crude OR 1.15; 95% CI 1.08 to 1.24; adjusted OR in Model 1 1.17; 95% CI 1.07 to 1.27; adjusted OR in Model 2 1.23; 95% CI 1.13 to 1.35).

### Association between GI and GDM

The influence of GI on the relationship between fruit consumption and GDM is also shown in Table 3. The increased consumption of fruit with moderate to high GI values was significantly associated with a higher likelihood of GDM. Compared with the lowest quartile, the highest quartile for consumption of fruits with moderate to high GI was associated with a higherlikelihood of GDM (crude OR 3.04; 95% CI 1.80 to 5.06; adjusted OR in Model 3, 2.94; 95% CI 1.47 to 5.88). Analyses conducted by using continuous variables yielded similar results, with an adjusted OR of 1.23 (95% CI 1.07 to 1.41) in Model 3. However, no significant association was observed between low GI fruit consumption and GDM.

### Association between subtypes of fruit according to polyphenol content and GDM

Comparison of fruit subtypes revealed that a greater consumption of pome fruit was associated with a lower likelihood of GDM (crude OR 0.59; 95% CI 0.37 to 0.96). The OR of GDM in the highest tertile of pome consumption was almost half that in the lowest tertile. However, the association attenuated to null after adjusting for potential confounding factors in Models 1, 2 and 3. Compared with the lowest tertile, the second tertile for consumption of gourd fruit was inversely associated with the likelihood of GDM, but this inverse association was neither observed in the highest tertile nor in the overall trend (P trend = 0.346). The adjusted ORs in Model 3 across the lowest to highest tertiles of fruit consumption were 1.00 (referent), 0.27 (95% CI 0.11 to 0.66) and 0.94 (95% CI 0.45 to1.95), respectively. In contrast, compared with the corresponding lowest tertiles, the highest tertiles for consumption of citrus and tropical fruit were each related to a higher likelihoodof GDM (adjusted OR in Model 3, 2.26; 95% CI 1.29 to 3.99; adjusted OR in Model 3, 3.73; 95% CI 1.74 to 8.01, respectively). Berry consumption was initially positively associated with GDM, but this association was attenuated to null in Model 3 (highest vs. lowest tertile in Model 3: OR, 1.69; 95% CI 0.80 to 3.56). Initially no significant association was observed between drupe consumption and GDM. However, a positive association was found after further adjustment for GI value and consumption of other fruit subgroups (adjusted OR in Model 3, 2.40; 95% CI 1.10 to 5.26) (Table 4). The results using continuous variables were almost consistent with those fitted as tertiles.

## Discussion

This was the first prospective study to specifically investigate the association between fruit consumption during pregnancy and GDM. The findings revealed that the excessive consumption of fruit during the second trimester may be associated with an increased likelihood of GDM. An increase in the consumption of fruit with moderate and high GI values, but not low GI values, was significantly associated with an elevated likelihood of GDM. Comparison of fruit subtypes revealed that greater consumption of tropical and citrus fruits was associated with a higher likelihood of GDM.

To the best of our knowledge, no previous studies have specifically examined the association between fruit consumption during pregnancy and the occurrence of GDM. However, two prospective studies were performed as part of the NHS II to investigate the effects of pre-pregnancy fruit consumption on the development of GDM^13^,^18^. One of these studies, which lasted for 8 years and involved 13,110 female nurses in the US, indicated that fruit fibre played a protective role in GDM^18^. However, no association was found between habitual pre-pregnancy fruit consumption and GDM risk in the other paper, a cohort study involving 13,475 female US nurses^13^. In addition, previous studies of fruit consumption and T2D risk have yielded mixed conclusions^9^,^10^,^11^,^12^. In some cases, greater fruit consumption was found to reduce T2D risk^16^,^19^,^20^,^21^,^22^, in others, no significant association was discovered^12^,^23^,^24^,^25^. The results of this study suggest that the excessive intake of fruit during the second trimester of pregnancy increases the occurrence of GDM.

Fruit has long been considered a protective factor in a range of diseases, as it has both a high antioxidant and fibre content and a relatively low energy density and GI^26^. However, fruit has also been found to contain relatively high levels of fructose, which is linked with insulin resistance and the impaired function of pancreatic β-cells^7^,^8^. We identified three plausible reasons for the positive relationship between fruit consumption and the occurrence of GDM. First, the overall health effect of fruit is determined by a combination of many bioavailable compounds in the fruit; therefore, a high fructose content may counteract the protective effect of fibre and other anti-diabetic compounds. A review indicated that fructose consumption may contribute to the development of obesity and the metabolic abnormalities that accompany insulin resistance^27^. Second, the mean fruit consumption (419 g/d)of the participants in this study exceeded the recommended daily intake (200 to 400 g/d) for pregnant women in China which may explain the positive association observed between fruit consumption and the occurrence of GDM^28^. A recent study revealed that the mean fruit consumption of pregnant women in Guangdong Province was 459.75 g/d, indicating that women in this province tend to consume fruit to excess during pregnancy^4^. In this study, mean fruit consumption in the third and fourth quartiles exceeded the upper limit of recommended daily intake, but the participants in the second quartile were within the recommended range. Compared with the lowest quartile group, in which mean consumption was below the lower limit of the recommended daily intake, the likelihood of GDM consistently increased in both the third and the fourth quartiles, but not in the second quartiles. We inferred that not only too little but too much fruit consumption increases the occurrence of GDM. Third, fruit type may influence the association between total fruit consumption and the likelihood of GDM. For example, GI value and polyphenol content, which have been suggested to be related to blood-glucose metabolism, differ substantially between fruit types^14^,^15^.

GI, a measure of the effects of carbohydrates on blood-glucose concentration, recently emerged as an important tool in diabetes management^29^,^30^,^31^. Some studies have been performed to determine whether the consumption of fruits with different GI values is associated with different probabilities of developing GDM. In a case-control study conducted in Guangdong Province, high GI fruit consumption during pregnancy was found to increase the GDM risk^4^. In addition, a clinical trial conducted in Toronto revealed that an increase in the consumption of low-GI fruit improved glycaemic control amongst people with T2D^32^. Consistent with these results, we found that the consumption of moderate-GI and high-GI fruit increased the occurrence of GDM, but not low-GI fruits. In another prospective study, however, no significant relationship was found between low GI fruit consumption and T2D risk, but greater consumption of moderate-GI fruit was related to a lower risk of T2D^16^. Therefore, the influence of fruit types with different GI values on the occurrence of GDM should be explored further in future research.

Various subtypes of fruit are considered an important dietary source of polyphenols, differing from individual compounds^33^. Although a number of related animal experiments – both *in vivo* and *in vitro* – have been performed to explore the biological mechanisms that underlie the hypoglycaemic effects of polyphenols, especially their individual constituents, scant research has been conducted on the association between fruit subtypes classified by polyphenol content and the risk of GDM^15^. To date, only one study has been designed to examine the effects of different fruit subtypes categorised according to polyphenol content on death of cardiovascular disease: the UK Women’s Cohort Study^17^. To help fill this research gap, we examined the influence on the occurrence of GDM of six subgroups of fruit categorised according to polyphenol content.

Pome fruits, which mainly comprise apples and pears, were the subgroup most frequently consumed by the study’s subjects, with a mean intake of 166 g/d. No previous studies have been performed to estimate the effect of pome intake on GDM, but some researchers have suggested that apple consumption protects against diabetes. The results of a previous large-scale prospective study indicated that total pre-pregnancy fruit consumption was not associated with GDM, but the authors recommended that pregnant women consume apples to prevent the development of GDM^13^. In another study, based on data from the NHS (n = 121,700), the NHS II (n = 116,671) and the Health Professionals Follow-up Study (n = 51,529), an inverse association was found between apple consumption and T2D risk^12^. Two studies with large samples (n = 38,018 and 10,054, respectively) indicated that dietary flavonoid in apples reduced the risk of T2D^34^,^35^. In our study, the consumption of pome fruit was again found to be inversely associated with the likelihood of GDM although the association attenuated to null after further adjustment for confounders. The main polyphenolic compounds in pome fruit are flavanols and hydroxycinnamic acids, similar to the polyphenol content of drupe fruit. However, greater drupe consumption was associated with a higher likelihood of GDM in the present study. Combined with the analysis in the previous study in which the association of apples consumption intake with GDM remained significant after adjustment for flavonoids intakes, we speculated that the protective effect of pome may not be attributed to polyphenols, but to low GI source of carbohydrate, other antioxidants or other unknown dietary factors^13^.

The intake of both citrus fruit and tropical fruit was found to increase the occurrence of GDM in this study. Although citrus fruits have been reported to contain numerous nutrients that help to guard against diabetes, such as flavanones, carotenoids, fibre and minerals (e.g. potassium and magnesium), a recent meta-analysis found no significant association between citrus fruit consumption and T2D risk^36^. The findings of our study even suggest that higher citrus intake may be positively associated with the likelihood of GDM. Therefore, the appropriate consumption of citrus fruit for women during pregnancy requires further investigation. No consensus has been reached on a formal scientific definition of ‘tropical fruit’. Although the word ‘tropical’ implies cultivation in the tropics, many fruits originally found in the tropics are now cultivated throughout the world. Compared with fruits grown in temperate regions of the world, tropical fruits have been studied in much less detail – especially those from Asia. Both tropical fruit and gourd fruit have been reported to contain lower levels of polyphenols and higher energy levels than other subtypes of fruit^33^,^37^. This may lead to the recommendation that pregnant women control their intake of tropical fruit. To date, little evidence has been obtained on the effects of gourd fruit consumption on glucose metabolism. Although a recent prospective study indicated that cantaloupe consumption is positively associated with GDM risk^16^, no association was found between gourd fruit intake and the occurrence of GDM in our study.

Some studies have indicated that the consumption of berries containing predominantly anthocyanidins can reduce the risk of T2D^12^,^16^,^22^. In addition, the results of a clinical trial suggested that the increased consumption of berries improved glycaemic control amongst people with diabetes^32^. In the present study, there was a significant association between berry intake and the occurrence of GDM. However, it became non-significant after adjustment for GI and other fruit subgroups consumption. This finding may be explained by the participants’ relatively low consumption of berries, as the concentration of active compounds in the berries consumed may not have been high enough to exert mechanistic effects *in vivo*. More studies are needed to investigate the association between anthocyanidin intake derived from fruit and the occurrence of GDM. Considering all the evidence given above, we did not have enough evidence to show that the difference in associations between different subtypes of fruit and GDM was due to different polyphenol contents.

Our study has certain strengths. First, it is a prospective study and thus provides strong evidence of causal relationships. Next, to the best of our knowledge, it is the first prospective study to specifically investigate not only the association between fruit consumption during pregnancy and the likelihood of GDM, but the effects on the occurrence of GDM of fruit polyphenol profile.

The study also had some limitations. First, the sample was smaller than that used in other large-scale prospective studies, due to implementation constraints. Second, 31.4% of the participants were lost during follow-up, mainly due to the high frequency of population movement in Dongguan City^37^. However, further investigation revealed no significant differences between the baseline characteristics of the study’s cohort and those of the participants lost to follow-up. This suggests that loss to follow-up had little influence on the results. Third, fruit consumption in Guangdong Province was found to be higher than the average daily intake in China^4^, reflecting the substantial variation in lifestyle, food availability and dietary habits across regions of China^38^. In addition, the GDM incidence in the population under study (21.9%) was higher than that in nationally representative populations of Chinese pregnant women (16%). Therefore, the generalizability of our findings was limited. Fourth, the dietary data collected in this study did not represent the participants’ diet in the long term, i.e., before pregnancy, which may affect the occurrence of GDM. However, due to the conspicuous changes in diet during the second trimester, it is appropriate to explore the short-term effects on the likelihood of GDM of fruit consumption during that time. In addition, although a single application of food records certainly is not the best method to reflect intake of a longer period, 3-day food records supported by face-to-face and telephone interviews for the estimation of dietary intake across the second trimester is satisfactory. Fifth, it is generally recognised that all self-reported methods of dietary assessment are extremely vulnerable to both random and systematic errors. To investigate the accuracy of the dietary intake data, we calculated the ratio of energy intake (EI) obtained in our study to the estimated energy requirement (EER) for China. The ratio of EI to EER was 0.97, which fell within the acceptable reported range of 0.76 to 1.24^39^. Sixth, although we controlled for a multitude of lifestyle and dietary factors in the multivariable analysis, the findings may still have been skewed by residual or unmeasured confounding factors. Seventh, the categorization of fruit into subgroups according to the Phenol Explorer database provides only a rough estimation of the polyphenolic profile. To date, however, Phenol-Explorer is the most comprehensive Web-based database on polyphenol content in foods, collating data from 638 high-quality articles published in peer-reviewed journals^33^.

In conclusion, the findings of this study suggest that the excessive consumption of fruit during the second trimester of pregnancy, especially moderate- and high-GI fruit, citrus fruit and tropical fruit, increases the occurrence of GDM. Further studies with larger sample sizes are needed to examine the extent to which subgroups of fruit divided by GI or polyphenol profile are associated with the occurrence of GDM.

## Methods and Materials

### Study subjects

This prospective study was conducted at the Dongguan Maternity and Child Health Care Hospital, Guangdong Province, China. The study commenced in April 2013 and was completed in August 2014. Its aim was to investigate the health consequences of selected dietary risk factors for pregnant women. Baseline data were collected in the first trimester of gestation (mean ± standard deviation [SD], 9.40 ± 2.14 weeks), and outcome data were obtained between 24 and 28 weeks of gestation (mean ± SD, 26.25 ± 1.33 weeks).

**Figure 1:**
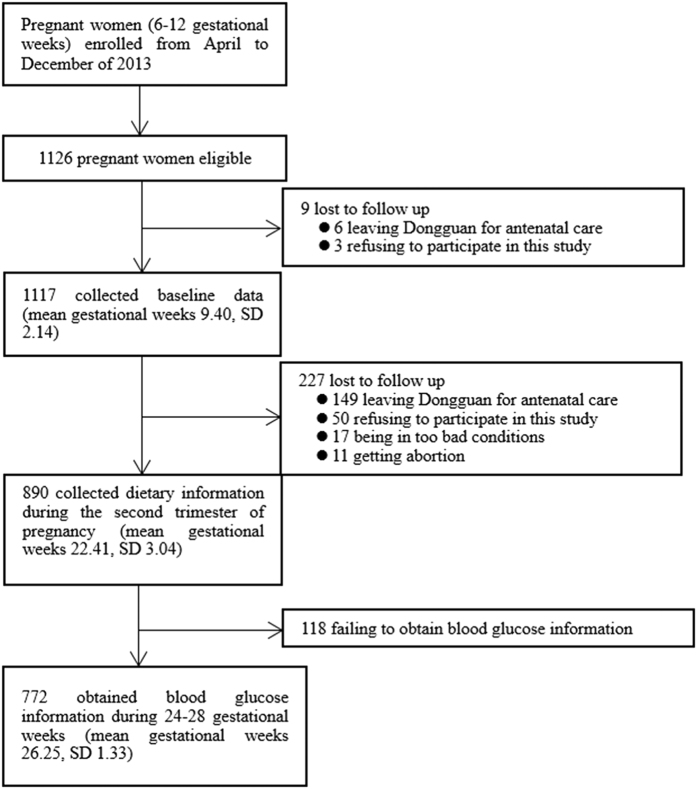
A flow chart for study participants in the cohort.

The participants were pregnant women registered at the Dongguan Maternity and Child Health Care Hospital in the first trimester of pregnancy (6 to 12 gestational weeks) from April to December 2013. Only primiparous women 20 to 35 years of age were considered eligible. Subjects were excluded if they had a history of childbearing or abortion at more than 4 months; exhibited a multiple pregnancy; had had diabetes (type 1 or type 2) or hypertension; or presented with renal insufficiency or kidney stones, thyroid-gland dysfunction, chronic obstructive pulmonary disease or asthma, the human immunodeficiency virus or active tuberculosis virus, mental disorders or anaemia. Of the cohort registered at the health care hospital in the period under study, 1126 women met the criteria for inclusion. During the follow-up period in the first and second trimesters of gestation, 354 women dropped out of the study for a variety of reasons. As a result, the data obtained from 772 women were subjected to analysis (Fig. 1).

The study was approved by the Biomedical Ethical Committee of Chinese Nutrition Society (CNS-2012-002). Written informed consent was obtained from all of the participants before the interviews, and the research methods were carried out in accordance with approved guidelines.

### Dietary assessment

Longitudinal face-to-face interviews were conducted by trained interviewers three times during the follow-up period: during the first trimester (≤12 weeks), the second trimester (13 to 27 weeks) and the last trimester (≥28 weeks) of gestation. Each subject was asked to complete a 3-day food record (2 weekdays and 1 weekend day), which was reported in a face-to-face interview on the first day and by telephone interview 2 days later. The participants were required to provide information on the types of food they had eaten each day and how much of each type they had eaten. A commonly used portion size was specified for each food type (e.g. a glass, a slice or a unit such as one apple or banana). To reduce measurement error, photographs of normal portions were provided to help the subjects to estimate and record their food consumption in the face-to-face interviews. The types and amounts of food were classified and coded based on the Chinese Food Composition Table^40^. The average daily consumption of all fresh fruit items reported by each participant was summed to calculate the total consumption of fresh fruit during the second trimester of pregnancy.

Individual fruits were categorised into three groups based on their GI values: low was defined as GI ≤ 55, moderate as GI > 55 & <70 and high as GI ≥ 70. Because the consumption of fruit in the moderate- and high-GI groups was too small to be analysed separately, fruits with moderate and high GI values were merged into a single group, defined as GI > 55^41^. Of the fruit consumed frequently by the participants, apples, pears, oranges, tangerines, grapefruits, peaches/nectarines, apricots, plums and strawberries were defined as low-GI fruit, and bananas, cantaloupes, melons, watermelons, pineapples and lychees were defined as moderate- and high-GI fruit^41^. In addition, following Lai *et al*., the above fruit types were divided into six subgroups according to their polyphenol content using the Phenol-Explorer database. These subgroups comprised pome fruit, citrus fruit, berries, drupe fruit, gourd fruit and tropical fruit (Table 5)^17^,^32^. Tropical fruit was defined as fruit cultivated in the tropics, such as bananas, mangoes and persimmons.

### Assessment of non-dietary covariates

Given the hypothesised association between fruit consumption and the occurrence of GDM, the following potential confounders were considered: age, education, occupation, income level, pre-pregnancy BMI, gestational weight gain, family history of diabetes, smoking status and alcohol use. Regular smokers were defined as those who had smoked at least one cigarette a day for more than 6 consecutive months; passive smoking was defined as exposure to the tobacco smoke of others for at least 5 minutes per day during the past 5 years; and regular drinking was defined as drinking alcohol at least once a week during the past year. Exercise level was assessed based on self-reported exercise activities for health. Gestational weight gain was calculated from the difference between weight before delivery and pre-pregnancy weight.

### Measurement of GDM

A standard 75 g 2 hour oral glucose tolerance test was used to diagnose GDM between 24 and 28 weeks of gestation at the Dongguan Maternity and Child Health Care Hospital. After overnight fasting for at least 8 hours, the women ingested a 250 ml solution containing 75 g glucose powder in the morning, and venous blood was drawn at fasting and 1 hour and 2 hours after the glucose load. All of the blood samples were tested at the laboratory of the Dongguan Maternity and Child Health Care Hospital. GDM was diagnosed using the criteria proposed by the International Association of Diabetes and Pregnancy Study Groups, namely any one of the following cut-off values: fasting plasma glucose (PG) level of 5.1 mmol/L or higher; a 1-hour PG level of 10.0 mmol/L or higher; or a 2-hour PG level of 8.5 mmol/L or higher^42^.

### Statistical analysis

The association between fruit consumption during the second trimester and the occurrence of GDM was investigated by multivariable logistic regression, providing odds ratios (ORs) and 95% confidence intervals (CIs). We chose this approach rather than the Cox proportional hazard regression because the participants’ person years, calculated by measuring the time from the beginning of the second trimester (usually the 13^th^ gestational week) to the date of GDM diagnosis (usually between the 24^th^ and the 28^th^ weeks), were similar. Quartiles of total fruit consumption were defined based on total fruit consumption of the whole cohort during the second trimester. A small amount of data on fruit consumption was missing; a lack of response was taken to indicate zero consumption. Tests for trends were performed using the median fruit intake of each quartile as continuous variables. Additionally, separate analyses were performed to estimate the association of fruit consumption grouped by the GI or polyphenolic profile with GDM. Fruit consumption in polyphenol subgroups was categorized into tertiles instead of quartiles, because too few subjects reported fruit consumption in polyphenol subgroups to allow for categorization into quartiles. Fruit consumption was also analysed as a continuous variable per 100 g/d increment.

We estimated the ORs and the 95% CIs using the following modelling strategy, based on already-known confounding variables. All models including crude OR were adjusted for energy intake using the energy-partitioning model^43^. For total fruit consumption, the energy intake from all non-fruit food groups was included as a covariate in the models. For fruit consumption grouped by the GI or polyphenolic profile, the specific energy intake from other fruit groups and non-fruit food groups was included as covariates. Model 1 was adjusted for the following covariates: age, education, occupation, income level, pre-pregnancy BMI, gestational weight gain, family history of diabetes, smoking status and alcohol use. Model 2 was adjusted for the variables in Model 1 plus the consumption of grain (continuous), vegetables (continuous), meat (continuous) and fish (continuous). For estimation of the association between different GI fruit and GDM, Model 3 was adjusted for the variables in Model 2 plus the consumption of fruit with other GI values. For estimation of the association between subtypes of fruit and GDM, Model 3 was adjusted for the variables in Model 2 plus GI value of other fruit subgroups (continuous) and the consumption of other subtypes of fruit (categorical). GI value was calculated as follows: the average GI of each fruit subgroup was multiplied by the amount of available carbohydrate in that fruit consumed (g/day). This product for all fruit subgroups was then summed and divided by the total intake of available carbohydrate^44^. Education was divided into the following groups: elementary/none, junior high school, high school, junior college and college. Occupation was categorised as white-collar, blue-collar, farmer/other or housewife/retired. Income level was divided into the following groups: less than 1000, 1000 to 3000, 3001 to 5000, 5001 to 10,000 and more than 10,001yuan/month. Pre-pregnancy BMI (kg/m) was calculated as the ratio of weight (kg) to squared height (m) and was categorised into four groups (<18.5, 18.5 to 23.9, 24 to 27.9 and ≥28 kg/m^2^)^45^.

For the baseline data, analysis of variance and the Kruskal-Wallis test were performed to test differences in the continuous variables, and chi-square and Fisher exact tests were used to analyse the discrete variables. All statistical analysis was conducted using SPSS 13.0 (SPSS Inc., Chicago, IL, USA). *P* values of less than 0.05 in a two-tailed test were considered to indicate statistical significance.

## Additional Information

**How to cite this article:** Huang, W.-Q. *et al*. Excessive fruit consumption during the second trimester is associated with increased likelihood of gestational diabetes mellitus: a prospective study. *Sci. Rep.* **7**, 43620; doi: 10.1038/srep43620 (2017).

**Publisher's note:** Springer Nature remains neutral with regard to jurisdictional claims in published maps and institutional affiliations.

## Figures and Tables

**Table 1:** Comparison of the baseline characteristics of thefinal cohort and the population lost to follow-up.

	The final cohort	The population lost to follow-up	*p* value^a^
	(n = 772)	(n = 354)	
Age (years)	26.01 ± 3.18	25.93 ± 3.34	0.08
Pre-pregnancy BMI (kg/m^2^)	19.74 ± 2.45	19.72 ± 2.31	0.07
Education			<0.001
Elementary/none	2 (0.3)	2 (0.6)	
Junior high school	85 (11)	86 (24.5)	
High school	190 (24.6)	97 (27.5)	
Junior college	230 (29.8)	94 (26.3)	
College	265 (34.3)	75 (21.1)	
Occupation			<0.001
White-collar worker	253 (32.8)	101 (28.4)	
Blue-collar worker	275 (35.6)	106 (30)	
Farmer/other	48 (6.2)	36 (10.3)	
Housewife/retired	196 (25.4)	111 (31.3)	
Income level (yuan/month)			0.03
<1000	7 (0.9)	7 (2)	
1000-	83 (10.8)	57 (16.2)	
3001-	265 (34.3)	119 (33.5)	
5001-	305 (39.5)	132 (37.3)	
10,001-	112 (14.5)	39 (11)	
Exercise (yes)	191 (24.7)	90 (25.4)	0.94
Smoking (yes)	21 (2.7)	60 (1.7)	0.28
Alcohol (yes)	20 (2.6)	60 (1.7)	0.28

Continuous variables are shown as means ± SDs, and categorical variables are shown as n(percentages). ^a^Chi-square test for categorical variables and Student *t*test for continuous variables.

**Table 2:** Baseline characteristics of women in different fruit consumption quartiles.

	Quartiles of fruit consumption				
	Q1	Q2	Q3	Q4	*p*value^a^
Age (years)	25.73 ± 3.11	26.09 ± 3.12	26.24 ± 3.16	26.73 ± 3.3	0.03
Gestational weight gain (kg)	14.11 ± 2.77	14.25 ± 2.22	14.34 ± 2.65	14.45 ± 2.67	0.611
Dietary factors					
Energy (kcal/d)	1603 (1365, 1958)	1671 (1457, 2019)	1817 (1600, 2246)	2579 (2116, 3338)	<0.001
Carbohydrate (g/d)	216 (178, 268)	227 (197, 284)	259 (219, 316)	389 (303, 493)	<0.001
Protein (g/d)	58 (44, 72)	59 (48, 71)	67 (51.1, 86)	87 (71, 126)	<0.001
Fat (g/d)	60 (50, 71)	58 (51, 69)	66 (57, 78)	86 (65, 104)	<0.001
Fruit (g/d)	182 (133, 206)	285 (266, 316)	425 (383, 471)	710 (601, 870)	<0.001
Grain (g/d)	272 (223, 331)	272 (232, 331)	290 (248, 364)	418 (306, 525)	<0.001
Vegetables (g/d)	220 (162, 300)	250 (180, 324)	228 (170, 318)	326 (205, 555)	<0.001
Meat (g/d)	107 (68, 163)	106 (75, 144)	126 (80, 190)	180 (120, 279)	<0.001
Fish (g/d)	33 (0, 80)	33 (0, 79)	36 (0, 100)	53 (0, 113)	0.005
Education					0.029
Elementary/none	0 (0.0)	1 (0.5)	1 (0.5)	0 (0)	
Junior high school	30 (15.5)	20 (10.4)	23 (11.9)	12 (6.2)	
High school	53 (27.3)	44 (22.9)	50 (25.9)	43 (22.3)	
Junior college	45 (23.2)	69 (35.9)	61 (31.6)	55 (28.5)	
College	66 (34.0)	58 (30.2)	58 (30.1)	83 (43.0)	
Occupation					0.789
White-collar worker	63 (32.5)	66 (34.4)	64 (33.2)	60 (31.1)	
Blue-collar worker	68 (35.1)	68 (35.4)	67 (34.7)	72 (37.3)	
Farmer/other	8 (4.1)	15 (7.8)	10 (5.2)	15 (7.8)	
Housewife/retired	55 (28.4)	43 (22.4)	52 (26.9)	46 (23.8)	
Income level (yuan/month)					0.145
<1000	2 (1.0)	2 (1.0)	1 (0.5)	2 (1.0)	
1000-	28 (14.4)	21 (10.9)	17 (8.8)	17 (8.8)	
3001-	81 (41.8)	65 (33.9)	62 (32.1)	57 (29.5)	
5001-	62 (32.0)	78 (40.6)	84 (43.5)	81 (42.0)	
10, 001-	21 (10.8)	26 (13.5)	29 (15.0)	36 (18.7)	
Pre-pregnancy BMI					0.246
<18.5	78 (40.2)	60 (31.3)	53 (27.5)	57 (29.5)	
18.5-	107 (55.2)	125 (65.1)	127 (65.8)	124 (64.2)	
24-	7 (3.6)	7 (3.6)	11 (5.7)	10 (5.2)	
28-	2 (1.0)	0 (0.0)	2 (1.0)	2 (1.0)	
Exercise (yes)	43 (22.2)	49 (25.5)	45 (23.3)	54 (28.0)	0.562
Smoking (yes)	7 (3.6)	4 (2.1)	6 (3.1)	4 (2.1)	0.732
Alcohol drinking (yes)	5 (2.6)	5 (2.6)	6 (3.1)	4 (2.1)	0.938
Family history of diabetes (yes)	23 (11.8)	16 (8.3)	20 (10.4)	28 (14.5)	0.203

Continuous variables were shown as mean ± SD or medians (P_25_, P_75_), and categorical variables were shown as n (percentages). ^a^Chi-square test or Fisher exact test for categorical variables, and analysis of variance or the Kruskal-Wallis test for continuous variables.

**Table 3:** Odds ratio and 95% confidence intervals of GDM in relation to the consumptions of total fruit and different GI fruit during the second trimester of gestation among participants from the final cohort.

	Fruit quartiles					
	Q1 (referent)	Q2	Q3	Q4	*p* trend^f^	Continuous (Per 100 g/d)
Total fruit						
Cases/Noncases, n	24/170	20/172	51/142	74/119		169/603
Intake, median (P_25_, P_75_) (g/d)	133 (183, 207)	285 (267, 317)	425 (383, 472)	710 (602, 870)		349 (233, 532)
Crude OR^a^	1	0.82 (0.44, 1.54)	2.37 (1.38, 4.05)	3.20 (1.83, 5.60)	<0.001	1.15 (1.08, 1.24)
Model 1^b^	1	0.93 (0.44, 1.96)	2.81 (1.47, 5.36)	3.47 (1.78, 6.36)	<0.001	1.17 (1.07, 1.27)
Model 2^c^	1	1.08 (0.50, 2.34)	3.03 (1.54, 5.94)	4.82 (2.38, 9.76)	<0.001	1.23 (1.13, 1.35)
Glycemic index						
Low						
Cases/Noncases, n	45/149	33/159	30/163	61/132		169/603
Intake, median (P_25_, P_75_) (g/d)	83 (34, 133)	200 (172, 202)	267 (247, 300)	457 (384, 553)		220 (144, 333)
Crude OR^d^	1	0.79 (0.47, 1.32)	0.65 (0.38, 1.09)	1.12 (0.69, 1.80)	0.871	1.01 (0.87, 1.19)
Model 1^b^	1	1.03 (0.56, 1.89)	0.92 (0.49, 1.73)	1.30 (0.74, 2.28)	0.434	1.04 (0.93, 1.17)
Model 2^c^	1	1.07 (0.58, 1.98)	0.95 (0.50, 1.79)	1.33(0.74, 2.37)	0.409	1.11 (0.98, 1.26)
Model 3^e^	1	1.18 (0.64, 2.25)	1.09 (0.57, 2.10)	1.61 (0.88, 2.94)	0.152	1.15 (1.01, 1.31)
Moderate or high						
Cases/Noncases, n	26/169	24/170	44/146	75/118		169/603
Intake, median (P_25_, P_75_) (g/d)	0 (0, 0)	36 (22, 50)	100 (82, 125)	258 (188, 383)		61 (10, 152)
Crude OR^b^	1	0.91 (0.50, 1.66)	1.75 (1.02, 3.00)	3.04 (1.80, 5.06)	<0.001	1.22 (1.10, 1.36)
Model 1^b^	1	0.76 (0.37, 1.54)	1.83 (0.96, 3.49)	2.57 (1.36, 4.86)	<0.001	1.22 (1.07, 1.38)
Model 2^c^	1	0.81 (0.39, 1.70)	1.99 (1.01, 3.92)	2.74 (1.39, 5.40)	<0.001	1.20 (1.05, 1.38)
Model 3^e^	1	0.81 (0.39, 1.72)	2.04 (1.03, 4.01)	2.94 (1.47, 5.88)	<0.001	1.23 (1.07, 1.41)

^a^Crude OR was adjusted for the energy intake from all non-fruit food groups according to the energy-partitioning model. ^b^Model 1 was adjusted for energy intake according to the energy-partitioning model, age, education, occupation, income level, pre-pregnancy BMI, gestational weight gain, family history of diabetes, smoking status and alcohol use. ^c^Model 2 was adjusted for the variables in Model 1 plus the consumption of grain, vegetables, meat and fish. ^d^Crude OR was adjusted for the energy intake from other fruit groups and non-fruit food groups according to the energy-partitioning model. ^e^Model 3 was adjusted for the variables in Model 2 plus the consumption of fruit with other GI values. ^f^*p*for linear trend obtained from models using the median intake of each quartile as continuous variables.

**Table 4:** Odds ratio and 95% confidence intervals of GDM in relation to different subtypes of fruit consumption during the second trimester of gestation among participants from the final cohort.

	Fruit tertiles			*p* trend^e^	Continuous (Per 100 g/d)
	T1(referent)	T2	T3		
Pome					
Cases/Noncases, n	66/200	62/255	41/148		169/603
Intake, medians (P_25_, P_75_) (g/d)	67 (0, 67)	167 (133, 200)	267 (267, 400)		133 (67, 200)
Crude OR^a^	1	0.78 (0.52, 1.17)	0.59 (0.37, 0.96)	0.030	0.86 (0.74, 0.99)
Model 1^b^	1	1.03 (0.64, 1.67)	0.57 (0.32, 1.00)	0.079	0.85 (0.72, 0.99)
Model 2^c^	1	1.09 (0.65, 1.81)	0.78 (0.43, 1.43)	0.493	0.94 (0.79, 1.13)
Model 3^d^	1	1.15 (0.65, 2.02)	0.86 (0.45, 1.64)	0.702	1.00 (0.84, 1.21)
**Citrus**					
Cases/Noncases, n	89/366	11/57	69/180		169/603
Intake, medians (P_25_, P_75_) (g/d)	0 (0, 0)	45 (27, 50)	167 (100, 250)		0 (0, 99)
Crude OR ^a^	1	1.14 (0.56, 2.30)	1.86 (1.27, 2.71)	0.002	1.38 (1.16, 1.65)
Model 1^b^	1	0.82 (0.34, 2.02)	2.09 (1.32, 3.32)	0.001	1.47 (1.18, 1.82)
Model 2^c^	1	0.84 (0.32, 2.17)	1.79 (1.10, 2.93)	0.013	1.35 (1.08, 1.69)
Model 3^d^	1	1.04 (0.37, 2.93)	2.26 (1.29, 3.99)	0.005	1.47 (1.16, 1.86)
**Berry**					
Cases/Noncases, n	42/236	43/194	84/173		169/603
Intake, medians (P_25_, P_75_) (g/d)	0 (0, 0)	17 (7, 27)	73 (50, 108)		14 (0, 50)
Crude OR ^a^	1	1.24 (0.77, 1.98)	2.10 (1.36, 3.25)	0.001	1.08 (0.77, 1.52)
Model 1^b^	1	1.04 (0.60, 1.81)	2.10 (1.24, 3.56)	0.002	1.21 (0.81, 1.81)
Model 2^c^	1	0.98 (0.55, 1.76)	2.44 (1.39, 4.29)	<0.001	1.41 (0.89, 2.23)
Model 3^d^	1	0.79 (0.40, 1.55)	1.69 (0.80, 3.56)	0.132	0.72 (0.40, 1.29)
**Drupe**					
Cases/Noncases, n	131/483	17/62	21/58		169/603
Intake, medians (P_25_, P_75_) (g/d)	0 (0, 0)	13 (12, 17)	83 (50, 150)		0 (0, 0)
Crude OR ^a^	1	1.01 (0.57, 1.79)	0.88 (0.54, 1.75)	0.672	1.15 (0.85, 1.57)
Model 1^b^	1	0.84 (0.41, 1.71)	1.12 (0.59, 2.11)	0.881	1.25 (0.89, 1.75)
Model 2^c^	1	1.16 (0.56, 2.43)	1.71 (0.86, 3.39)	0.133	1.66 (1.10, 2.51)
Model 3^d^	1	1.16 (0.52, 2.57)	2.40 (1.10, 5.26)	0.039	1.87 (1.20, 2.90)
Gourd					
Cases/Noncases, n	123/459	9/83	37/61		169/603
Intake, medians (P_25_, P_75_) (g/d)	0 (0, 0)	22 (11, 33)	133 (83, 267)		0 (0, 0)
Crude OR ^a^	1	0.49 (0.17, 1.46)	0.97 (0.63, 1.50)	0.781	1.21 (0.74, 1.42)
Model 1^b^	1	0.44 (0.14, 1.41)	1.17 (0.66, 2.10)	0.386	1.16 (0.96, 1.40)
Model 2^c^	1	0.38 (0.11, 1.31)	1.32 (0.70, 1.50)	0.505	1.17 (0.96, 1.42)
Model 3^d^	1	0.27 (0.11, 0.66)	0.94 (0.45, 1.95)	0.346	1.18 (0.95, 1.45)
Tropical fruit					
Cases/Noncases, n	34/224	40/223	95/156		169/603
Intake, medians (P_25_, P_75_) (g/d)	0 (0, 7)	53 (42, 80)	168 (133, 245)		6 (53, 133)
Crude OR ^a^	1	1.14 (0.69, 1.87)	3.22 (2.04, 5.08)	<0.001	1.44 (1.19, 1.75)
Model 1^b^	1	0.95 (0.53, 1.71)	3.14 (1.82, 5.40)	<0.001	1.41 (1.14, 1.74)
Model 2^c^	1	0.94 (0.51, 1.72)	3.34 (1.89, 5.91)	<0.001	1.50 (1.20, 1.87)
Model 3^d^	1	0.92 (0.44, 1.92)	3.73 (1.74, 8.01)	<0.001	1.69 (1.28, 2.24)

^a^Crude OR was adjusted for the energy intake from other fruit groups and non-fruit food groups according to the energy-partitioning model. ^b^Model 1 was adjusted for the energy intake according to the energy-partitioning model, age, education, occupation, income level, pre-pregnancy BMI, gestational weight gain, family history of diabetes, smoking status and alcohol use. ^c^Model 2 was adjusted for the variables in Model 1 plus the consumption of grain, vegetables, meat and fish. ^d^Model 3 was adjusted for the variables in Model 2 plus GI value of other fruit subgroups and the consumption of other subtypes of fruit. ^e^*p* for linear trend obtained from models using the median intake of each tertile as continuous variables.

**Table 5:** Subtypes of fruit categorised by polyphenol content.

Subtypes	Fruits	Major polyphenol composition
Pome fruit	Apple, pear	Flavanols, hydroxycinnamic acids, flavonols
Citrus fruit	Orange, tangerine, grapefruit	Flavanones
Berries	Strawberries, grapes	Anthocyanins, flavanols, hydroxybenzoic acids
Drupe fruit	Peach, nectarine, plum, apricot, cherries	Hydroxycinnamic acids, flavanols, anthocyanins
Gourd fruit	Cantaloupe, melon, watermelon	—
Tropical fruit	Banana, mango, persimmon, lichee, longan, papaya, pitaya, pineapple, kiwi, guava, loquat, jackfruit	—
